# Discovery of Pre-Treatment FDG PET/CT-Derived Radiomics-Based Models for Predicting Outcome in Diffuse Large B-Cell Lymphoma

**DOI:** 10.3390/cancers14071711

**Published:** 2022-03-28

**Authors:** Russell Frood, Matthew Clark, Cathy Burton, Charalampos Tsoumpas, Alejandro F. Frangi, Fergus Gleeson, Chirag Patel, Andrew F. Scarsbrook

**Affiliations:** 1 Department of Nuclear Medicine, Leeds Teaching Hospitals NHS Trust, Leeds LS2 9JT, UK; chirag.patel13@nhs.net (C.P.); a.scarsbrook@nhs.net (A.F.S.); 2 Department of Radiology, Leeds Teaching Hospitals NHS Trust, Leeds LS2 9JT, UK; matt.clark1@nhs.net; 3 Leeds Institute of Health Research, University of Leeds, Leeds LS9 7TF, UK; 4 Department of Haematology, Leeds Teaching Hospitals NHS Trust, Leeds LS2 9JT, UK; cathy.burton1@nhs.net; 5 Department of Nuclear Medicine and Molecular Imaging, University Medical Center of Groningen, University of Groningen, 9713 AV Groningen, The Netherlands; c.tsoumpas@rug.nl; 6 Leeds Institute of Cardiovascular and Metabolic Medicine, University of Leeds, Leeds LS2 9JT, UK; a.frangi@leeds.ac.uk; 7 Centre for Computational Imaging and Simulation Technologies in Biomedicine (CISTIB), School of Computing and School of Medicine, University of Leeds, Leeds LS2 9JT, UK; fergus.gleeson@ouh.nhs.uk; 8 Medical Imaging Research Center (MIRC), University Hospital Gasthuisberg, Katholieke Universiteit Leuven, 3000 Leuven, Belgium; 9 Department of Radiology, Oxford University Hospitals NHS Foundation Trust, Oxford OX3 9DU, UK

**Keywords:** diffuse large B-cell lymphoma, lymphoma, predictive modelling, radiomics, machine learning

## Abstract

**Simple Summary:**

Diffuse large B-cell lymphoma (DLBCL) is the most common type of lymphoma. Even with the improvements in the treatment of DLBCL, around a quarter of patients will experience recurrence. The aim of this single centre retrospective study was to predict which patients would have recurrence within 2 years of their treatment using machine learning techniques based on radiomics extracted from the staging PET/CT images. Our study demonstrated that in our dataset of 229 patients (training data = 183, test data = 46) that a combined radiomic and clinical based model performed better than a simple model based on metabolic tumour volume, and that it had a good predictive ability which was maintained when tested on an unseen test set.

**Abstract:**

Background: Approximately 30% of patients with diffuse large B-cell lymphoma (DLBCL) will have recurrence. The aim of this study was to develop a radiomic based model derived from baseline PET/CT to predict 2-year event free survival (2-EFS). Methods: Patients with DLBCL treated with R-CHOP chemotherapy undergoing pre-treatment PET/CT between January 2008 and January 2018 were included. The dataset was split into training and internal unseen test sets (ratio 80:20). A logistic regression model using metabolic tumour volume (MTV) and six different machine learning classifiers created from clinical and radiomic features derived from the baseline PET/CT were trained and tuned using four-fold cross validation. The model with the highest mean validation receiver operator characteristic (ROC) curve area under the curve (AUC) was tested on the unseen test set. Results: 229 DLBCL patients met the inclusion criteria with 62 (27%) having 2-EFS events. The training cohort had 183 patients with 46 patients in the unseen test cohort. The model with the highest mean validation AUC combined clinical and radiomic features in a ridge regression model with a mean validation AUC of 0.75 ± 0.06 and a test AUC of 0.73. Conclusions: Radiomics based models demonstrate promise in predicting outcomes in DLBCL patients.

## 1. Introduction

Diffuse large B-cell lymphoma (DLBCL) is the commonest subtype of non-Hodgkin lymphoma (NHL), accounting for approximately 30–40% of adult cases [1]. The gold standard treatment is immunochemotherapy with rituximab, cyclophosphamide, doxorubicin hydrochloride, vincristine (Oncovin) and prednisolone (RCHOP) [2]. Radiotherapy can be added if there is bulky or residual disease. Prophylactic intrathecal methotrexate or intravenous treatment with chemotherapy that crosses the blood-brain barrier may be included if there is high risk for central nervous system (CNS) involvement [3]. Even with current therapy regimes, approximately 20–30% of patients will recur following treatment [4,5]. Staging and response assessment is performed using 2-deoxy-2-[fluorine18]-fluoro-D-glucose (FDG) positron emission tomography/computed tomography (PET/CT). Treatment stratification based on mid-treatment (interim) PET/CT is commonly used in the management of patients with Hodgkin lymphoma but is less established in DLBCL due to the reduced ability to accurately predict treatment outcome in this lymphoma subtype mid-treatment [6,7]. There is increasing interest in the use of PET/CT derived metrics for treatment stratification at baseline in lymphoma to improve patient outcome. A number of groups have explored the potential utility of baseline metabolic tumour volume (MTV) for predicting event free survival (EFS) with promising results, but this has yet to be adopted clinically [8,9,10,11,12,13,14,15,16,17]. Others have explored the potential utility of radiomic features extracted from PET/CT for modelling purposes [8,18]. Initial results are promising, however, the published studies with relatively small numbers of patients are heterogenous

This aim of this study was to develop and test models combining baseline clinical information and radiomic features extracted from PET/CT imaging in DLBCL patients to predict 2-year EFS (2-EFS) using data from our tertiary centre. The secondary aim was to compare model performance to the predictive ability of baseline MTV.

## 2. Materials and Methods

The transparent reporting of a multivariable prediction model for individual prognosis or diagnosis (TRIPOD) guidelines were adhered to as part of this study (Supplementary Material).

### 2.1. Patient Selection

Radiological and clinical databases were retrospectively reviewed to identify patients who underwent baseline PET/CT for DLBCL at our institution between January 2008 and January 2018. A cut-off of January 2018 was chosen to allow a minimum of 2 years follow up without interference or confounding factors introduced by the COVID-19 pandemic. Patients were excluded if they did not have DLBCL, were under 16 years of age, had no measurable disease on PET/CT, had hepatic involvement, had a concurrent malignancy, were not treated with R-CHOP or if the images were degraded or incomplete. A 2-EFS event was defined as disease progression, recurrence or death from any cause within the 2-year follow up period.

### 2.2. PET/CT Acquisition

All imaging was performed as part of routine clinical practice. Patients fasted for 6 h prior to administration of intravenous Fluorine-18 FDG (4 MBq/kg). PET acquisition and reconstruction parameters for the four scanners used at our institution are detailed in Table 1. Attenuation correction was performed using a low-dose unenhanced diagnostic CT component acquired using the following settings: 3.75 mm slice thickness; pitch 6; 140 kV; 80 mAs.

### 2.3. Image Segmentation

All PET/CT images were reviewed and contoured using a specialised multimodality imaging software package (RTx v1.8.2, Mirada Medical, Oxford, UK). FDG-positive disease segmentation was performed by either a clinical radiologist with six years’ experience or a research radiographer with two years’ experience. Contours were then reviewed by dual-certified Radiology and Nuclear Medicine Physicians with >15 years’ experience of oncological PET/CT interpretation. Any discrepancies were agreed by consensus.

Two different semi-automated segmentation techniques were used. The first applied a fixed standardised uptake value (SUV) threshold of 4.0, and the second used a threshold derived from 1.5 times mean liver SUV. The 4.0 SUV threshold was selected based on previous work assessing different segmentation techniques in a cohort of DLBCL patients by Burggraaff et al. which found it had a higher interobserver reliability [19] and requires less adaption than techniques such as 41% SUVmax. The 1.5 times mean liver SUV threshold was chosen as an adaptive threshold technique which has been used in different cancer types [20,21], and allows for adaptive thresholding which takes into consideration background SUV uptake which can vary between individuals. Mean liver SUV was calculated by placing a 110 cm^3^ spherical region of interest (ROI) in the right lobe of the liver. The PET image contour was translated to the CT component of the study with the contours matched to soft tissue with a value of −10 to 100 Hounsfield units (HU). Contours were saved and exported as digital imaging and communications in medicine (DICOM) radiotherapy (RT) structures. Both the images and contours were converted to Neuroimaging Informatics Technology Initiative (NIfTI) files using the python library Simple ITK (v2.0.2) (https://simpleitk.org/, accessed on 1 December 2021).

### 2.4. Feature Extraction

Feature extraction was performed using PyRadiomics (v2.2.0) (https://pyradiomics.readthedocs.io/en/latest/index.html, accessed on 1 December 2021). Both the CT and PET images were resampled to a uniform voxel size of 2 mm^3^. Radiomic features were extracted from the entire segmented disease for each patient. A fixed bin width of 2.5 HU was used for the CT component. Two different bin-widths were used when extracting the radiomic features from the PET component. The first being derived by finding the contour with the maximum range of SUVs and dividing this by 130, the second being derived by dividing the maximum range by 64. This methodology was based on previous work by Orlhac et al. and on PyRadiomics documentation [22]. The first and second order features were extracted from both the PET and CT components. Further higher order features were explored by extracting the first and second order features following application of wavelet, log-sigma, square, square root, logarithm, exponential, gradient and local binary pattern (lbp)-3D filters to the images. All the features extracted and the filters applied are detailed in Table S1. The mathematical definition of each of the radiomic features can be found within the PyRadiomics documentation [23]. PyRadiomics deviates from the image biomarker standardisation initiative (IBSI) by applying a fixed bin width from 0 and not the minimum segmentation value, and the calculation of first order kurtosis being +3 [24,25]. Otherwise, PyRadiomics adheres to IBSI guidelines. Patient age, disease stage and sex were also included as clinical features in the models. Disease stage and sex were dummy encoded using Pandas (v1.2.4) (https://pandas.pydata.org/pandas-docs/stable/whatsnew/v1.2.4.html, accessed on 1 December 2021). This resulted in a total of 3935 features extracted per patient. ComBat harmonisation was applied to account for the different scanners used within the study (https://github.com/Jfortin1/ComBatHarmonization, accessed on 1 December 2021) [26].

### 2.5. Machine Learning

The dataset was split into a training and test set stratified around 2-EFS, disease stage, age and sex with an 80:20 split using scikit-learn (v0.24.2) (https://scikit-learn.org/stable/whats_new/v0.24.html, accessed on 1 December 2021). Concordance between the demographics of the training and test groups was assessed using a *t*-test for continuous data and a χ^2^ test for categorical data. A *p*-value of <0.05 was regarded as significant. Continuous data was normalised using a standard scaler (scikit-learn v0.24.2) which was trained and fit on the training set and subsequently applied to the test set. Highly correlated features were removed from the training and test sets if they had a Pearson coefficient over 0.8. This reduced the number of features from 3935 down to 130 for each patient.

Six different machine learning (ML) classifiers were used: logistic regression with lasso, ridge and elasticnet penalties, support vector machine (SVM), random forest and k-nearest neighbour. A maximum number of five features were included within each model, apart from in the lasso and elasticnet models where these classifiers determined the optimum number of features. To avoid false discoveries (Type 1 errors), a maximum number of five features was chosen guided by the rule of 1 feature per 10 events within the training set. Feature selection for the remaining models was performed using three different methods: a forward wrapper method (mlxtend 0.18.0), a univariate analysis method (scikit-learn v0.24.2), and a recursive feature extraction method (where applicable) (scikitlearn v0.24.2). Each method was used to create a list of features from two to the maximum five features which were to be explored in the training phase. The features selected were based on the highest mean receiver operating characteristic (ROC) curve area under the curve (AUC) in a four-fold stratified cross validation with 25 repeats.

**Figure 1:**
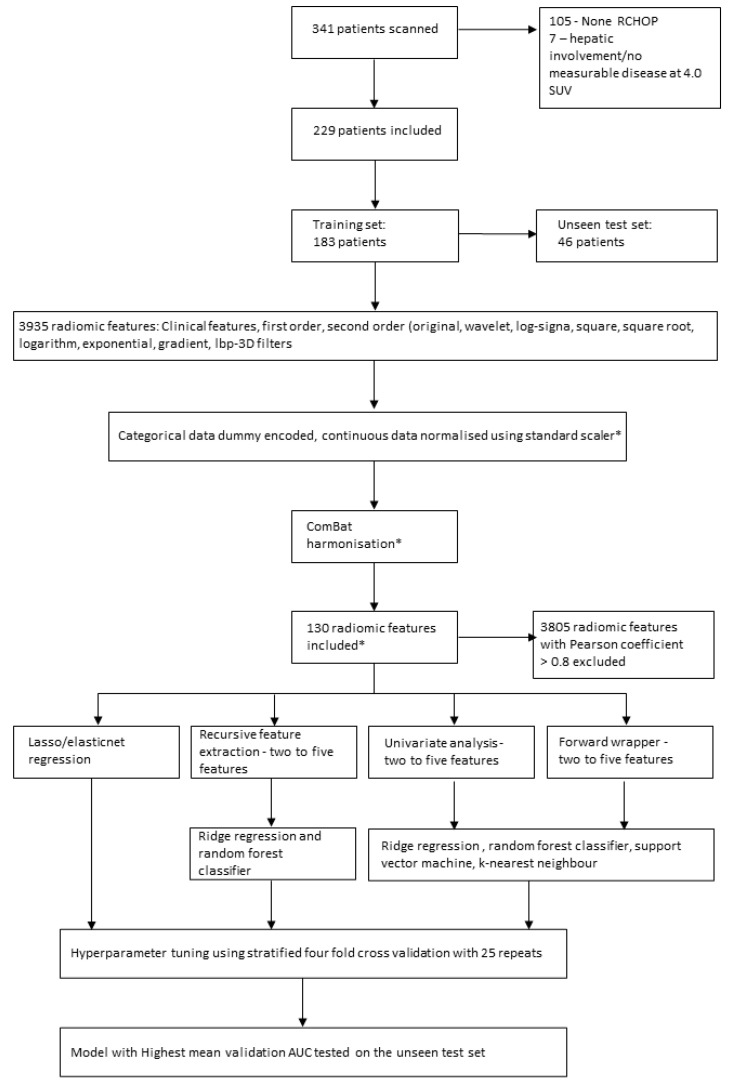
Pathway for patient inclusion, feature selection and model creation. * = initially applied to the training data and then to the test data.

Training of the ML models and the tuning of hyperparameters was performed using a grid search with a stratified four-fold cross validation stratified around 2-EFS with 25 repeats. The list of hyperparameters explored within the grid search are detailed in Table S2. Features and hyperparameters with the highest mean validation AUC which was within 0.05 of the mean training AUC were selected. A 0.05 cut-off was chosen to try and minimise selection of an overfitted model. The model which had the highest mean validation AUC overall was tested once on the unseen test set. The Youden index was used to discover the optimum cut-off value from the ROC curve and the accuracy, sensitivity, specificity, negative predictive value (NPV) and positive predictive value (PPV) were calculated from this for the unseen test set. The pipeline for patient inclusion, feature selection and predictive model creation and testing is depicted in Figure 1.

Given the growing evidence surrounding MTV as a predictor of outcome, two further logistic regression models were derived from the MTVs using the different segmentation. A comparison between results from the different cross validation splits between the radiomic model with the mean highest AUC and the MTV model with the mean higher AUC was performed using a Wilcoxon signed ranked test.

## 3. Results

A total of 229 DLBCL patients met the inclusion criteria (136 male, 93 female) with 62 2-EFS events. There were 183 patients within the training cohort and 46 patients in the unseen test cohort. No statistically significant differences were identified between the training and test sets (Table 2).

None of the machine learning models created using elasticnet regression, lasso regression or k-nearest neighbour algorithms had a mean validation AUC within 0.05 of the mean training AUC. The remaining model results are presented in Table 3 and Table 4.

**Figure 2:**
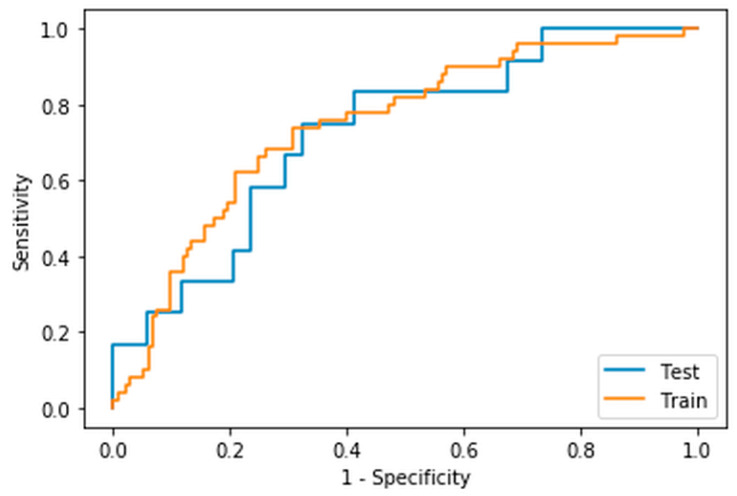
ROC Curve of the training and unseen test data AUCs for the model derived using a 4.0 SUV thresholding segmentation technique with a bin width derived from SUVmax/64.

The model within the highest mean validation ROC AUC was the ridge regression model created using radiomic features extracted from a fixed threshold of 4.0 SUV segmentation using a bin width of the maximum range of SUVs divided by 64. The mean training AUC was 0.77 ± 0.02, the mean validation AUC was 0.75 ± 0.06 and the AUC when tested on the unseen dataset was 0.73 (Figure 2). The features selected with their coefficients and intercept are presented in Table 5. A threshold of 0.5 was chosen and led to an accuracy of 0.70, sensitivity of 0.44, specificity of 0.86, positive predictive value of 0.67, and a negative predictive value of 0.71. The confusion matrix is presented in Table 6.

**Figure 3:**
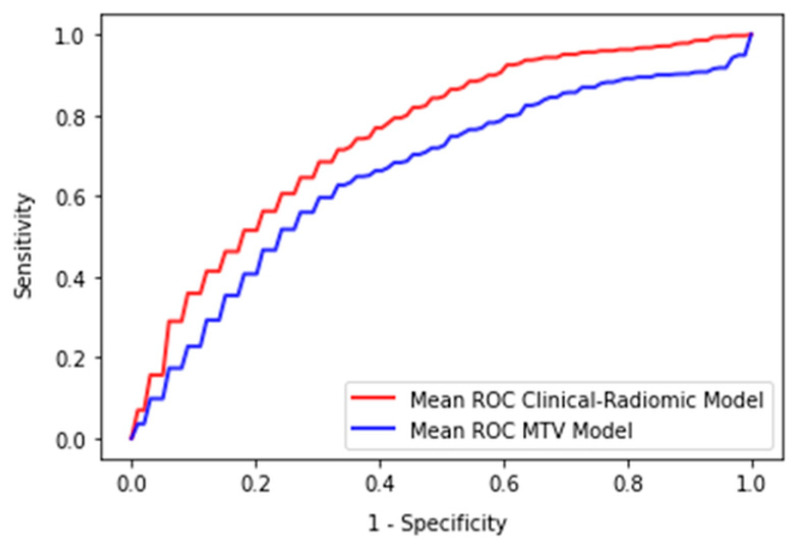
Mean ROC Curve of the MTV-based logistic regression model and the radiomic-based logistic regression model.

The logistic regression model created solely from MTV using the 4.0 SUV fixed threshold segmentation technique had a mean training AUC of 0.66 ± 0.03 and a mean validation AUC of 0.66 ± 0.08. The logistic regression model derived from MTV using the 1.5 times mean liver SUV segmentation technique had a mean training AUC of 0.67 ± 0.03 and a mean validation AUC of 0.67 ± 0.08. There was a statistically significant difference when comparing the cross validation AUCs for the 100 splits between the highest performing MTV-based model and the radiomic-based ridge regression model, *p* < 0.001 (Figure 3).

## 4. Discussion

Our study found that a prediction model combining clinical and radiomic features derived from pretreatment PET/CT using a ridge regression model had the highest mean validation AUC when predicting 2-EFS in DLBCL patients. This model had significantly higher validation AUCs than those achieved by a model solely derived from MTV and achieved an AUC of 0.73 on the unseen test set. The radiomic features used within the model that led to the highest mean validation AUC were extracted from a segmentation derived from a fixed threshold of 4.0 SUV using a bin-width calculated from the maximum range of SUVs divided by 64. The model was formed using five features (Stage Four, PET original GLSZM large area emphasis, PET wavelet-HHL GLSZM Small Area Emphasis, PET wavelet-HHH GLSZM Grey Level Non-Uniformity normalised, PET square 10th percentile).

The biological correlate of radiomic features and how these relate to the lesion or disease process can often be overlooked, and can become more complex when image filtering is involved [27]. Three of the radiomic features included in the best model were derived from GLSZM which is a matrix formed by the number of connected voxels with the same grey level intensity. The first was the PET GLSZM Large Area Emphasis, which is a measure of distribution of large area size zones, and was extracted from the PET data without any filter applied. This feature is higher in lesions which have a coarser texture based on the original image. The other two GLZMs are calculated after applying a wavelet filter. Wavelet filters highlight or suppress certain spatial frequencies within an image. In PyRadiomics a combination of high and low filters is passed in each of the different dimensions, which results in eight different decompositions. PET wavelet-HHL GLSZM Small Area Emphasis is a measure of the distribution of small size zones, which are higher in lesions with fine textures following the application of the wavelet filter. PET wavelet-HHH GLSZM Grey Level Non-Uniformity is a measure of the variability of the grey level intensity within the image. A lower value indicates a higher number of similar SUVs on the wavelet filtered image. The last radiomic feature included was PET square 10th percentile which is the 10th percentile value of the SUV after a square of the image SUVs has been taken and normalised to the original SUV range. Interestingly, none of the CT-derived radiomic features were selected as part of the best performing radiomic models. This is likely due to the transposition of the segmentations from the PET on to the unenhanced CT including more areas of non-lymphomatous tissue.

Other studies which have explored the use of radiomic features in outcome prediction in DLBCL are not always directly comparable [12,28,29,30,31,32]. This is mainly due to differences in segmentation methodology, modelling techniques and outcome measures between groups. Aide et al. studied the use of radiomic features in predicting 2-EFS in 132 patients (training = 105, validation = 27) and found that MTV as well as four second-order metrics and five third-order metrics were selected from ROC analyses. However, long-zone high-grey level emphasis was the only independent predictor when analysed with the international prognostic index (IPI) and MTV [29]. In our study long-zone high-grey level emphasis was discarded when checking for multicollinearity. This highlights a potential issue of radiomic model development when applying a methodology on different datasets. It may be that the same features would be chosen between the different datasets, but each method removes the alternate correlated feature and, therefore, appears to create an entirely new model. Both Zhang et al. and Ceriani et al. used lasso in their cox regression models to select the most appropriate features [31,32]. Zhang et al. in a study of 152 patients (training = 100, validation = 52) treated with R-CHOP or R-EPOCH (rituximab, etoposide, prednisone, vincristine, cyclophosphamide, and doxorubicin) found that a survival model created with radiomic features and MTV had a validation time dependent ROC AUC of 0.748 (95% CI 0.596–0.886). A model created with radiomic features and metabolic bulk volume had a validation time dependent ROC AUC 0.759 (95% CI 0.595–0.888). Ceriani et al. reported that a radiomic score derived from a training set of 133 patients and tested on an external dataset of 107 patients had an AUC of 0.71 in both the test and validation datasets. The features selected within their cox regression model were GLCM sum squares, maximum 3D diameter and GLDM grey level variance, GLSZM grey level non-uniformity normalised.

In our study both lasso and elasticnet methods failed to produce a model that achieved mean training and validation scores within 0.05 of each other. Even when allowing for a more generous difference between the training and validation scores, mean validation scores remained below 0.65. This 0.05 cut-off is arbitrary and was applied to try and reduce the impact of overfitting on the dataset and allow selection of a potentially more generalisable model. Despite this, there is still a risk that both training and validation datasets are overfitted and the model would need external validation on an external dataset.

One of the largest published studies by Decazes et al. in 215 DLBCL patients, explored use of tumour volume surface ratio and total tumour surface as outcome predictors for 5-year progression free survival (PFS), but found that MTV outperformed both features with MTV having an AUC of 0.67 [12]. This AUC for MTV is similar to the findings in our study, with the mean validation AUC for MTV prediction of 2-EFS being 0.66 for the 4.0 SUV threshold and 0.67 for the 1.5 times liver threshold segmentation techniques, respectively. Although, there is growing interest in the use of MTV as an imaging biomarker, Adams et al. reported, in a study of 73 DLBCL patients, that the prognostic ability of MTV does not add anything to the prognostic ability of the clinical scoring system National Comprehensive Cancer Network-International Prognostic Index (NCCN-IPI) [33]. Unfortunately, due to missing clinical data it was not possible to compare IPI performance in our patient cohort. However, this does highlight the potential impact of confounders on the generalisability of predictive models. Although, causality is not generally considered in predictive modelling, its use in future models could allow for greater transparency of a model. The issues of generalisability may be compounded by learnt biases towards groups of patients in the training process.

The TRIPOD checklist was completed to increase transparency of model development [34,35]. However, there are limitations to our study including its retrospective nature and uncertainty surrounding the exact timing and recording of recurrence. Use of 2-EFS partially mitigates against this by allowing a wider window for the relapse to be recorded, however, it does mean that data which could have been included in a time to survival type model is lost. 2-EFS was chosen as the majority of patients relapse within the first 2 years. Time to event ML models could be used in future studies to reduce the need to exclude data. The lesions were not re-segmented as part of the study, and therefore, calculations of inter or intra-reliability, as well as robustness of the features have not been performed. ComBat harmonization was used to help mitigate against scanner variation in the extracted feature extraction. However, this limits the ability to apply this model prospectively to patients not scanned using a protocol used to train the model. Lack of clinical data surrounding the IPI and cell of origin (COO) information, meant that these could not be used as direct comparators to radiomic models created.

## 5. Conclusions

A combined clinical and PET/CT derived radiomics model using ridge regression demonstrated the highest mean AUC validation (AUC = 0.75) when predicting 2-EFS in DLBCL patients treated with R-CHOP, which outperformed a model derived solely from MTV (AUC = 0.67).

## Figures and Tables

**Table 1:** Reconstruction parameters for the different scanners used.

Scanner	Voxel Size in mm (x, y, z)	Matrix	Reconstruction	Scatter Correction	Randoms Correction
Philips Gemini TF64	4 × 4 × 4	144 or 169	BLOB-OS-TF	SS-Simul	DLYD
GE Healthcare Discovery 690	3.65 × 3.65 × 3.27	192	VPFX	Model based	Singles
GE Healthcare Discovery 710	3.65 × 3.65 × 3.27	192	VPFX	Model based	Singles
GE Healthcare STE	4.6875 × 4.6875 × 3.27	128	OSEM	Convolution subtraction	Singles

BLOB-OS-TF = an ordered subset iterative TOF reconstruction algorithm using blobs instead of voxels; DLYD = delayed event subtraction; OSEM = ordered subsets expectation maximisation; SS-Simul = single-scatter simulation; VPFX = Vue Point FX (OSEM including point spread function and time of flight).

**Table 2:** Demographics of the training and testing groups.

Demographic	Training Cohort	Test Cohort	*p*-Value
Age	67 (IQR = 17)	65 (IQR = 22.5)	0.35
Sex			
Male	107	29	0.69
Female	76	36	
Radiotherapy			
Yes	78	20	0.95
No	105	26	
Stage			
One	42	17	0.26
Two	46	6	
Three	31	6	
Four	64	17	
2-EFS Event			
Yes	50	12	0.98
No	133	34	

2-EFS = 2-year event free survival. The *p*-values were calculated using a *t*-test for age and a χ^2^ test for the remaining demographic features.

**Table 3:** Mean training and validation scores for the best performing machine learning models using the 4.0 SUV threshold segmentation technique.

Machine Learning Model	Hyperparameters	Features	AUC Mean Training	AUC Mean Validation
SUVmax/130				
Ridge Regression	C: 1 × 10^−5^, penalty: l2, solver: liblinear	Stage One, PET wavelet-LLH GLSZM Large Area Emphasis, PET wavelet-HHH GLSZM Grey Level Non-Uniformity Normalised, PET square 10th Percentile, PET square GLDM Grey Level Non-Uniformity	0.75 (0.02)	0.74 (0.07)
Support Vector Machine	C: 1, gamma: 0.008915428868611115, kernel: sigmoid	PET wavelet-HHH GLSZM Grey Level Non-Uniformity Normalised, PET square 10th Percentile, PET lbp-3D-m1 Interquartile Range, PET lbp-3D-m1 GLDM Large Dependence Low Grey Level Emphasis, PET lbp-3D-k 90th Percentile	0.74 (0.02)	0.73 (0.07)
Random Forest	bootstrap: False, max depth: 1, max features: log2, min samples leaf: 50, min samples split: 50, n estimators: 10	PET original shape Maximum 2D Diameter Column, MTV, PET original first order Kurtosis, PET original GLSZM Large Area Emphasis, PET wavelet-LHL GLCM Correlation, PET wavelet-LHL GLCM Imc2	0.76 (0.02)	0.71 (0.08)
**SUVmax/64**				
Ridge Regression	C: 0.001, penalty: l2, solver: newton-cg	Stage Four, PET original GLSZM Large Area Emphasis, PET wavelet-HHL GLSZM Small Area Emphasis, PET wavelet-HHH GLSZM Grey Level Non-Uniformity Normalised, PET square 10th Percentile	0.77 (0.02)	0.75 (0.06)
Support Vector Machine	C: 0.1, gamma: 0.07938667031015477, kernel: rbf	PET original GLDM Large Dependence Low Grey Level Emphasis, PET wavelet-HHH GLSZM Grey Level Non-Uniformity Normalised, PET square 10th Percentile, PET lbp-3D-k 90 Percentile, PET lbp-3D-k GLSZM Size Zone Non-Uniformity Normalised	0.75 (0.02)	0.72 (0.06)
Random Forest	bootstrap: True, max depth: 1, max features: log2, min samples leaf: 44, min samples split: 6, n estimators: 243	PET original shape Maximum 2D Diameter Column, PET original shape Surface Volume Ratio, PET original 10th Percentile	0.71 (0.02)	0.69 (0.08)

l2 = Ridge regression penalty, liblinear = A library for large linear classification, GLSZM = grey level size zone matrix, GLDM = grey level dependence matrix, lbp-3D-m1 = local binary pattern filtered image at level 1, lbp-3D-k = local binary pattern kurtosis image, GLCM = grey level co-occurrence matrix, rbf = radial basis function.

**Table 4:** Mean training and validation scores for the best performing machine learning models using the 1.5 times mean liver SUV thresholding segmentation technique.

Machine Learning Model	Hyperparameters	Features	AUC Mean Training	AUC Mean Validation
SUVmax/130				
Ridge Regression	C: 1 × 10^−5^, penalty: l2, solver: saga	Stage Four, Age, PET original GLDM Large Dependence Low Grey Level Emphasis, PET original GLSZM Large Area High Grey Level Emphasis	0.74 (0.03)	0.71 (0.09)
Support Vector Machine	C: 1, gamma: 0.43727367418726576, kernel: rbf	PET square 10th Percentile, PET square first order Energy	0.78 (0.02)	0.73 (0.07)
Random Forest	bootstrap: True, max depth: 10, max features: sqrt, min samples leaf: 33, min samples split: 5, n estimators: 90	Age, PET original shape Elongation, PET original shape Least Axis Length, PET original shape Major Axis Length, PET original shape Maximum 2D Diameter Column, PET original shape Mesh Volume		
**SUVmax/64**				
Ridge Regression	C: 1.0, penalty: l2, solver: liblinear	Stage Three, Age, PET wavelet-LHL GLCM Imc1, PET square GLDM Dependence Variance, PET square GLSZM Small Area Low Grey Level Emphasis	0.76 (0.02)	0.73 (0.07)
Support Vector Machine	C: 1, gamma: 0.43727367418726576, kernel: rbf	PET square first order 10 Percentile, PET square first order Energy	0.78 (0.02)	0.73 (0.07)
Random Forest	bootstrap: True, max depth: 10, max features: log2, min samples leaf: 42, min samples split: 6, n estimators: 237	PET original shape Sphericity, PET original GLSZM Large Area Emphasis	0.70 (0.02)	0.69 (0.07)

l2 = Ridge regression penalty, liblinear = A library for large linear classification, GLSZM = grey level size zone matrix, GLDM = grey level dependence matrix, lbp-3D-m1 = local binary pattern filtered image at level 1, lbp-3D-k = local binary pattern kurtosis image, GLCM = grey level co-occurrence matrix, rbf = radial basis function.

**Table 5:** **The** features selected and their associated coefficients and intercept in the ridge regression model tested on the unseen test dataset.

Feature	Coefficient
Stage Four	0.01153414
PET original GLSZM Large Area Emphasis	0.0161316
PET wavelet-HHL GLSZM Small Area Emphasis	0.01482446
PET wavelet-HHH GLSZM Grey Level Non-Uniformity Normalised	−0.01923886
PET square 10 Percentile	−0.01923886
Intercept	−0.01166859

**Table 6:** Confusion matrix for the threshold of 0.5.

Prediction	Negative	Positive
Predicted Negative	24	10
Predicted Positive	4	8

Positive = recorded 2-EFS event, Negative = no recorded 2-EFS event, Predicted Positive = predicted to have had a 2-EFS event, Predicted Negative = predicted to not have had a 2-EFS event.

## Data Availability

The data are not publicly available due to institutional data sharing restrictions.

## Supplementary Materials

The following supporting information can be downloaded at: https://www.mdpi.com/article/10.3390/cancers14071711/s1, TRIPOD Checklist: Prediction Model Development, Table S1: Radiomic features extracted for both the PET and CT components, Table S2: The hyperparameters explored within the grid search.
