# Supplementary material for: Discovery of Pre-Treatment FDG PET/CT-Derived Radiomics-Based Models for Predicting Outcome in Diffuse Large B-Cell Lymphoma
Source: Cancers (Basel). 2022 Mar 28;14(7):1711. doi: 10.3390/cancers14071711 (PMC8997127; doi:10.3390/cancers14071711)
Supplement: Supplementary file 1 [file cancers-14-01711-s001.zip › cancers-1649080-supplementary.pdf]

Supplemental Material

TRIPOD Checklist: Prediction Model Development

Section/Topic	Item	Checklist Item	Page
**Title and abstract**			
Title	1	Identify the study as developing and/or validating a multivariable prediction model, the target population, and the outcome to be predicted.	*
Abstract	2	Provide a summary of objectives, study design, setting, participants, sample size, predictors, outcome, statistical analysis, results, and conclusions.	*
**Introduction**			
Background and objectives	3a	Explain the medical context (including whether diagnostic or prognostic) and rationale for developing or validating the multivariable prediction model, including references to existing models.	*
	3b	Specify the objectives, including whether the study describes the development or validation of the model or both.	*
**Methods**			
Source of data	4a	Describe the study design or source of data (e.g., randomized trial, cohort, or registry data), separately for the development and validation data sets, if applicable.	*
	4b	Specify the key study dates, including start of accrual; end of accrual; and, if applicable, end of follow-up.	*
Participants	5a	Specify key elements of the study setting (e.g., primary care, secondary care, general population) including number and location of centres.	*
	5b	Describe eligibility criteria for participants.	*
	5c	Give details of treatments received, if relevant.	*
Outcome	6a	Clearly define the outcome that is predicted by the prediction model, including how and when assessed.	*
	6b	Report any actions to blind assessment of the outcome to be predicted.	*
Predictors	7a	Clearly define all predictors used in developing or validating the multivariable prediction model, including how and when they were measured.	*
	7b	Report any actions to blind assessment of predictors for the outcome and other predictors.	*
Sample size	8	Explain how the study size was arrived at.	*
Missing data	9	Describe how missing data were handled (e.g., complete-case analysis, single imputation, multiple imputation) with details of any imputation method.	*
Statistical analysis methods	10a	Describe how predictors were handled in the analyses.	*
	10b	Specify type of model, all model-building procedures (including any predictor selection), and method for internal validation.	*
	10d	Specify all measures used to assess model performance and, if relevant, to compare multiple models.	*
Risk groups	11	Provide details on how risk groups were created, if done.	*
**Results**			

Participants	13a	Describe the flow of participants through the study, including the number of participants with and without the outcome and, if applicable, a summary of the follow-up time. A diagram may be helpful.	*
	13b	Describe the characteristics of the participants (basic demographics, clinical features, available predictors), including the number of participants with missing data for predictors and outcome.	*
Model development	14a	Specify the number of participants and outcome events in each analysis.	*
	14b	If done, report the unadjusted association between each candidate predictor and outcome.	*
Model specification	15a	Present the full prediction model to allow predictions for individuals (i.e., all regression coefficients, and model intercept or baseline survival at a given time point).	*
	15b	Explain how to the use the prediction model.	*
Model performance	16	Report performance measures (with CIs) for the prediction model.	*
**Discussion**			
Limitations	18	Discuss any limitations of the study (such as nonrepresentative sample, few events per predictor, missing data).	*
Interpretation	19b	Give an overall interpretation of the results, considering objectives, limitations, and results from similar studies, and other relevant evidence.	*
Implications	20	Discuss the potential clinical use of the model and implications for future research.	*
**Other information**			
Supplementary information	21	Provide information about the availability of supplementary resources, such as study protocol, Web calculator, and data sets.	*
Funding	22	Give the source of funding and the role of the funders for the present study.	*

*See section in comments.

We recommend using the TRIPOD Checklist in conjunction with the TRIPOD Explanation and Elaboration document.

1 This study looks at the utility of pre-treatment FDG PET/CT derived machine learning models for outcome prediction in diffuse large B-cell lymphoma (DLBCL). (**Title**)

2 A summary of all the requested information is presented in the **Abstract.**

3 a) The introduction presents the background, aim of the model and the previous studies which have explored models containing radiomic features from the baseline PET/CT. (**Introduction**)

b) The aim of this study was to create a predictive model using both clinical and radiomic features derived from pre-treatment FDG PET/CT to predict 2-year EFS in DLBCL patients using a cohort from a tertiary treatment centre (**Introduction**)

4 a) Retrospective single centre cohort study. The study cohort was randomised on a ratio of 4:1 into training and testing cohorts stratified around 2-EFS, age, sex and disease stage. (**Patient selection**)

b) Consecutive patients with histologically proven DLBCL NOS who underwent baseline FDG-PET/CT at a single large tertiary referral centre between June 2008 and January 2018 were included. The follow up information recorded is set out in the patient selection section. (**Patient selection**)

5 a) This is a single tertiary centre study. (**Patient selection**)
b) Patients were excluded if they did not have DLBCL NOS, were under 16 years of age, had no measurable disease on PET/CT when using a 4.0SUV or had hepatic involvement, had a concurrent malignancy, were not treated with R-CHOP or if the images were degraded on incomplete. The follow up information recorded is set out (**Patient selection**)
c) The treatment regimen was with RCHOP for all patients. No change to departmental standard treatment was performed. (**Patient selection**)

6 a) A 2-EFS event was defined as recurrence or death from any cause within the 2-year follow up period. (Patient selection)
b) As this was a retrospective study the primary outcomes were defined from clinical records. The investigator reviewing the records was blinded to the imaging parameters.

7 a) The description of the contouring method, resampling, harmonisation, radiomic feature extraction, feature selection and model training and testing is documented within the materials and methods section. The features selected as part of the chosen model are described the results section. (**Materials and methods, Results**)
b) The images were contoured blinded to the outcome data.

8 All patients which met the inclusion criteria were included. The cut-off of January 2018 was chosen to allow for 2 years follow up whilst minimising confounding factors introduced by the Covid-19 pandemic. For feature selection 5 features were chosen as the maximum number of features to be include in each model. This was derived from 10 events per parameter, with 50 events within the training cohort. This was not the case for the lasso and elastinet models whose feature selection was derived by the penalty applied to the models (**Materials and methods, Results**)

9 Only complete data sets were used in the analysis. (**Results**)

10 a) Clinical factors were included in the variable selection process alongside radiomic features. The categorical data was dummy encoded and the continuous features were normalised using a standard scaler. (**Machine learning analysis**)
b) Random forest, support vector machine, ridge regression, lasso regression, elasticnet regression and k-nearest neighbour models were trained and tuned on the training cohort using four fold cross validation with 25 repeats. The models were created using different feature selection methods, using two different segmentation techniques and two different PET bin widths. The model with the highest mean receiver operating characteristic (ROC) area under the curve (AUC) was tested once on the unseen test cohort. (**Machine learning analysis**)
d) When comparing models, the mean validation AUC was used to determine the best performing model, the model with the highest being tested on the unseen test set (**Machine learning analysis**)

11 Risk groups were not created within the model.

13 a) 229 patients were included, with demographics detailed in **Table 2.** (**Results**)

b) The characteristics of the participants are presented in **Table 2**.

14 a) The number of events per cohort are presented in **Table 2**.
b) This has not been performed. The training and testing cohorts were stratified around key clinical features but the results are not adjusted for these.

15 a/b) The features and hyperparameters used to create the model are presented in the results section.

16 The mean validation and test ROC curves are presented, standard deviations are presented for the mean training and mean validation scores. The confusion matrix with the accuracy, sensitivity, specificity, negative predictive value and negative predictive value for the best performing model with the a threshold derived using the Youden index from the ROC curve (**Results**)

18 The limitations of the study are presented in the discussion. These include the retrospective nature of the study, the relative low number of events, reliance on clinical records, the exclusion of patients with hepatic disease or not having measurable disease above 4.0 SUV, and that there was no external validation. (**Discussion**)

19 b)/20. The discussion section gives an overall interpretation of the results, it highlights the potential use of a pre-treatment model, but discusses the next steps needed to make this clinically viable (**Discussion**)

21 The python libraries used are references within the text and the radiomic features extracted using PyRadiomics are detailed in **Supplementary Material Table 1**.

22 Individual author's funding is declared within the **Declaration**. The study was not externally funded.

Table S1. Radiomic features extracted for both the PET and CT components.

First Order	Shape	GLCM	GLRLM	GLDM	GLSZM	NGTDM
10th Percentile	Elongation	Autocorrelation	Grey Level Non-Uniformity	Dependence Entropy	Grey Level Non-Uniformity	Busyness
90th Percentile	Flatness	Cluster Prominence	Grey Level Non-Uniformity Normalized	Dependence Non-Uniformity	Grey Level Non-Uniformity Normalized	Coarseness
Energy	Least Axis Length	Cluster Shade	Grey Level Variance	Dependence Non-Uniformity Normalized	Grey Level Variance	Complexity
Entropy	Major Axis Length	Cluster Tendency	High Grey Level Run Emphasis	Dependence Variance	High Grey Level Zone Emphasis	Contrast

Inter quartile Range	Maximum 2D Diameter Column	Contrast	Long Run Emphasis	Grey Level Non-Uniformity	Large Area Emphasis	Strength
Kurtosis	Maximum 2D Diameter Row	Correlation	Long Run High Grey Level Emphasis	Grey Level Variance	Large Area High Grey Level Emphasis	
Maximum	Maximum 2D Diameter Slice	Difference Average	Long Run Low Grey Level Emphasis	High Grey Level Emphasis	Large Area Low Grey Level Emphasis	
Mean Absolute Deviation	Maximum 3D Diameter	Difference Entropy	Low Grey Level Run Emphasis	Large Dependence Emphasis	Low Grey Level Zone Emphasis	
Mean	Mesh Volume	Difference Variance	Run Entropy	Large Dependence High Grey Level Emphasis	Size Zone Non-Uniformity	
Median	Minor Axis Length	Id	Run Length Non-Uniformity	Large Dependence Low Grey Level Emphasis	Size Zone Non-Uniformity Normalized	
Minimum	Sphericity	Idm	Run Percentage	Low Grey Level Emphasis	Small Area Emphasis	
Range	Surface Area	Idmn	Run Variance	Small Dependence Emphasis	Small Area High Grey Level Emphasis	
Robust Mean Absolute Deviation	Surface Volume Ratio	Idn	Short Run Emphasis	Small Dependence High Grey Level Emphasis	Small Area Low Grey Level Emphasis	
Root Mean Squared	Voxel Volume	Imc1	Short Run High Grey Level Emphasis	Small Dependence Low Grey Level Emphasis	Zone Entropy	
Skewness		Imc2	Short Run Low Grey Level Emphasis		Zone Percentage	
Total Energy		Inverse Variance			Zone Variance	

Uniformity	Joint Average
Variance	Joint Energy
	Joint Entropy
	MCC
	Maximum Probability
	Sum Average
	Sum Entropy
	Sum Squares

The equations for the features can be found at https://pyradiomics.readthedocs.io/en/latest/features.html. GLCM = grey level co-occurrence matrix, GLDM = grey level dependence matrix, GLRLM = grey level run length matrix, GLSZM = grey level size zone matrix, NGTDM = neighbouring grey tone difference matrix, Id = inverse difference, Idn = inverse difference normalised, Imc = informational measure of correlation, Idm = inverse difference moment, Idmn = inverse difference moment normalised, MCC = maximal correlation coefficient. Each of the first and second order features were extracted from the original imaging and then from the images following filters applied. The filters used were: wavelet; log-signa; square; square root; logarithm; exponential; gradient; lbp-3D.

Table S2. The hyperparameters explored within the grid search and the hyperparameters kept static when training the different machine learning models.

Model	List of hyperparameters explored	Static hyperparameters
Lasso Logistic Regression	C = [0.0000001, 0.000001, 0.00001, 0.0001, 0.001, 0.01, 0.1, 1, 10, 100]<br>solver = ['liblinear', 'saga']	penalty = 'l1'<br>random_state=0<br>class_weight="balanced"<br>max_iter =10000
Elasticnet Logistic Regression	C = [0.0000001, 0.000001, 0.00001, 0.0001, 0.001, 0.01, 0.1, 1, 10, 100]<br>l1_ratio = [0.1, 0.2, 0.3, 0.4, 0.5, 0.6, 0.7, 0.8, 0.9]	penalty = 'elasticnet'<br>solver = "saga"<br>random_state=0, class_weight="balanced"<br>max_iter =10000
Ridge Logistic Regression	C = [0.0000001, 0.000001, 0.00001, 0.0001, 0.001, 0.01, 0.1, 1, 10, 100]<br>solver = ['liblinear', 'saga', "sag", 'lbfgs', 'newton-cg']	penalty = 'l2'<br>random_state=0<br>class_weight="balanced"<br>max_iter =10000
Support Vector Machine	C = [0.0000001, 0.000001, 0.00001, 0.0001, 0.001, 0.01, 0.1, 1, 10]<br>gamma = expon(scale=.1)<br>kernel = ['linear', 'rbf', "sigmoid", "poly"]	random_state=0 class_weight="balanced"<br>probability = True
Random Forest	n_estimators = [int(x) for x in np.linspace(start = 10, stop = 250, num = 40)]<br>max_features = ['log2', 'sqrt']<br>max_depth = [int(x) for x in np.linspace(start = 1, stop = 10, num = 5)]<br>min_samples_split = [int(x) for x in np.linspace(start = 2, stop = 50, num = 49)]<br>min_samples_leaf = [int(x) for x in np.linspace(start = 2, stop = 50, num = 49)]<br>bootstrap = [True, False]	random_state = 0<br>class_weight = "balanced"
K-Nearest Neighbour	n_neighbors = range(1, 21, 2)<br>weights = ['uniform', 'distance']<br>metric = ['euclidean', 'manhattan', 'minkowski']	

If a hyperparameter is not documented, it was left as the default within the library.
